# IL‐35 recombinant protein reverses inflammatory bowel disease and psoriasis through regulation of inflammatory cytokines and immune cells

**DOI:** 10.1111/jcmm.13428

**Published:** 2017-11-29

**Authors:** Yuan Wang, Ying Mao, Junfeng Zhang, Gang Shi, Lin Cheng, Yi Lin, Yiming Li, Xiaomei Zhang, Yujing Zhang, Xiaolei Chen, Jie Deng, Xiaolan Su, Lei Dai, Yang Yang, Shuang Zhang, Dechao Yu, Yuquan Wei, Hongxin Deng

**Affiliations:** ^1^ State Key Laboratory of Biotherapy and Cancer Center/Collaborative Innovation Center of Biotherapy West China Hospital Sichuan University Chengdu China; ^2^ Laboratory Animal Center Sichuan University Chengdu China

**Keywords:** IL‐35 recombinant protein, inflammatory bowel disease, psoriasis, immunoregulation

## Abstract

Interleukin‐35 (IL‐35), a member of the IL‐12 family, functions as a new anti‐inflammatory factor involved in arthritis, psoriasis, inflammatory bowel disease (IBD) and other immune diseases. Although IL‐35 can significantly prevent the development of inflammation in many diseases, there have been no early studies accounting for the role of IL‐35 recombinant protein in IBD and psoriasis. In this study, we assessed the therapeutic potential of IL‐35 recombinant protein in three well‐known mouse models: the dextransulfate sodium (DSS)‐induced colitis mouse model, the keratin14 (K14)‐vascular endothelial growth factor A (VEGF‐A)‐transgenic (Tg) psoriasis mouse model and the imiquimod (IMQ)‐induced psoriasis mouse model. Our results indicated that IL‐35 recombinant protein can slow down the pathologic process in DSS‐induced acute colitis mouse model by decreasing the infiltrations of macrophages, CD4^+^T and CD8^+^T cells and by promoting the infiltration of Treg cells. Further analysis demonstrated that IL‐35 recombinant protein may regulate inflammation through promoting the secretion of IL‐10 and inhibiting the expression of pro‐inflammatory cytokines such as IL‐6, TNF‐α and IL‐17 in acute colitis model. In addition, lower dose of IL‐35 recombinant protein could achieve long‐term treatment effects as TNF‐α monoclonal antibody did in the psoriasis mouse. In summary, the remarkable therapeutic effects of IL‐35 recombinant protein in acute colitis and psoriasis mouse models indicated that IL‐35 recombinant protein had a variety of anti‐inflammatory effects and was expected to become an effective candidate drug for the treatment of inflammatory diseases.

## Introduction

IL‐35 was proposed as an immunosuppressive factor in 2007 for the first time and together with IL‐12, IL‐23 and IL‐27 form the IL‐12 family 1, 2. IL‐35 is a heterodimer composed of p35 and EBI3 subunits and is preferentially secreted by mouse and human Treg cells 3, 4. IL‐35 is highly expressed in the bone marrow, thymus, blood and liver tissues of mice, but we can find its constitutive expression only in human trophoblast cells 5, 6. IL‐35 is able to induce the generation of Treg cells that produce IL‐35 (iTr35 cells) in mice and human 7. In contrast to the pro‐inflammatory effects of other IL‐12 family cytokines (IL‐12, IL‐23), IL‐35 potently inhibits the CD4^+^ effector T cells including Th1 and Th17 cells through the expansion of Treg cells as well as IL‐10 secretion 8, 9. Several diseases including multiple inflammatory diseases, coronary artery disease and cancer have been shown to be associated with increased IL‐35 expression 1, 5, 10. IL‐35 has become a potential therapeutic agent for immune‐related diseases. In the rheumatoid arthritis mouse model, IL‐35 could promote the secretion of IL‐10 and effectively improve the disease. Foundational work utilized *IL‐12a* ^*−/−*^and *Ebi3* ^*−/−*^ mice found Treg cells lacking IL‐35 expression had a significantly reduced ability to suppress T cell proliferation 11, 12, indicating the critical role of IL‐35 in immune regulation 2.

Inflammation, the protection of body against infection, could lead persistent damage to irreversible pathological changes in issues even including cancer if out of control 13, 14, 15. Therefore, it is essential to maintain the balance of immune homoeostasis through keeping the inflammatory response under precise and effective regulation. Inflammatory bowel diseases (IBD) is an autoimmune disease including ulcerative colitis (UC) and Crohn's disease (CD) whose pathogenesis has not been fully elucidated 16, 17. Recent studies have examined the concept that IBD could result from dysregulation of the intestinal barrier and a pathologic activation of the intestinal immune response towards several bacterial or viral antigens 18. Indeed, dysregulation of the intestinal immune response is a hallmark of chronic inflammatory conditions, and the disease may lead to colon cancer 12, 19. The need to find a suitable and effective treatment programme has attracted the attention of many medical workers.

Psoriasis is a highly recurrent skin immune disease characterized by skin lesions. The incidence in the world is about 0.1–3%, and 5 million people suffer from this disease in China 20, 21. The symptoms of psoriasis are mainly reflected in the epidermal proliferation and inflammatory cell infiltration, accompanied by rapid proliferation and differentiation of keratinocytes, over‐angiogenesis in dermal papillary as well as T cell infiltration 20, 22. The complex pathogenesis of psoriasis is a result of genetic inheritance, immune, infection and other related factors which still cannot be fully clarified 23. This intractable skin disease can lead to systemic damage which often needs lifelong treatment, seriously affecting the patients’ normal life. During the recent years, the treatment making use of immune cells and cytokines has been successfully applied to clinical remission of autoimmune diseases which provides inspiration for the development of new drugs in the treatment of psoriasis 24.

Previous studies have shown that compared with wild‐type mice, EBI3‐deficient mice were subjected to the worsen symptoms of acute colitis and significantly shorter survival, suggesting that IL‐35 may be involved in the regulation of the occurrence in colitis 11, 25. In addition, IL‐35 plays significantly immunomodulatory effects in a variety of systemic autoimmune diseases 26. We have demonstrated that IL‐35 plasmid‐based therapy could weaken the psoriasis inflammatory process by regulating inflammatory cytokine secretion and macrophage infiltration 27. However, the therapeutic effect may be impeded by the low transfection efficiency *in vivo* and inflammatory response caused by plasmid 28. Protein‐based therapeutics are highly successful in clinic and currently enjoy unprecedented recognition of their potential, in this context, recombinant protein‐based therapy may represent an alternative approach 29. There have been no earlier studies accounting for the role of IL‐35 recombinant protein in IBD and psoriasis. In our present study, we investigated the therapeutic effects of IL‐35 recombinant protein in DSS‐induced acute colitis mouse model, K14‐VEGF transgenic psoriasis mouse model and IMQ‐induced psoriasis mouse model.

The results of our study demonstrated that IL‐35 recombinant protein could inhibit the progression of inflammation and achieve significant therapeutic effects in acute colitis and psoriasis mouse model. Our results highlighted the critical role of IL‐35 recombinant protein in the regulation of immune microenvironment and provided new insights into the treatment of colitis, psoriasis as well as other inflammatory diseases.

## Materials and methods

### DSS‐induced colitis mouse model and therapy

Seven‐week‐old male C57BL/6J mice [Vital River Laboratories (VRL), Beijing, China] were housed in individual animal cages. After 5 days of acclimatization, the mice were assigned to six groups with equal mean body weight, and each group comprised of eight mice: (*i*) the blank group, which received sterilized tap water with tail vein injection (i.v.) during the experimental period; (*ii*) the PBS control group, which received the daily injections (i.v.) of PBS and colitis was induced by 2.5% (*w*/*v*) DSS‐containing sterilized tap water for 7 days; (*iii*) the hIL‐35‐Fc group, which received the daily injections (i.v.) of 2.5 μg hIL‐35‐Fc (Sino Biological Inc., Beijing, China) and colitis was induced by 2.5% (*w*/*v*) DSS‐containing sterilized tap water for 7 days. The therapeutic regime in colitis mouse mode is once a day for 6 days in total *via* the tail vein. All animal protocols were approved by the Animal Care and Use Committee of Sichuan University (Chengdu, China).

### K14‐VEGF‐A‐tg mouse psoriasis model and therapy

The female K14‐VEGF‐A‐Tg homozygous mice (The Jackson Laboratory, Bar Harbor, ME, USA) were gifts from Professor Jiong Li from State Key Laboratory of Biotherapy. K14‐VEGF‐A‐Tg mice overexpress VEGF in the epidermis, and these mice spontaneously develop a chronic inflammatory skin disease with many features similar to those seen in human psoriasis 30. K14‐VEGF‐A‐Tg homozygous mice were randomly assigned to two groups (*n* = 8 per group) with a total of ten injection (i.v.) of PBS, 2 or 5 μg hIL‐35‐Fc per mouse every other day. Thirty days or 60 days after the last dose, the animals were anaesthetized with 10% chloral hydrate (i.p.) for macroscopic photography. The therapeutic regimes of IBI303 [12.5 mg/kg body weight (about 325 μg per mouse), Innovent Biologics, Inc., Suzhou, China] in psoriasis mice have been described in our previous study 31. Briefly, the therapeutic regimes in psoriasis model are four cycles, the first therapy cycle is continuously for 5 days in the first week, then three times a week for the second cycle and twice a week for the third and fourth cycle. Mice were killed after IBI303 treatment for 4 weeks.

### IMQ‐induced psoriasis mouse model and therapy

BALB/c mice (8‐week‐old) received a daily topical dose of 62.5 mg commercially available 5% IMQ cream (Aldara; 3M Pharmaceuticals, Tallinn, Estonia) on a shaved area on the back for 6 days. This dose was empirically determined to cause optimal and reproducible skin inflammation in mice. Two weeks before this model was established, we administered (i.v.) PBS or 5 μg hIL‐35‐Fc per mouse as prophylactic treatment every other day for a total of seven times, subsequent three more injections were conducted *via* the tail vein during establishment of the model.

### Assessment of colitis symptoms

Mice were monitored daily for body weight loss. Colon bleeding score was assessed as the combined score of faecal consistency and blood in the stool throughout the DSS treatment and recovery period (scored as 0–4). Mice were killed, and the entire colon was removed from the caecum to the anus. The length of the colon was measured. Each 5‐μm‐thick slice of tissue was sectioned and stained by haematoxylin and eosin (H&E). Inflammation score graded the degree of inflammatory cell infiltrations and tissue damage, resulting in a combined score ranging from 0 (no changes) to 4 (widespread cellular infiltrations and extensive tissue damage).

### Flow cytometry

The spleen and lymph node (LN) of treated mice were collected and suspended as single cells with red blood cell lysis buffer. Colorectal tissues from treated mice were collected and suspended as single cells with collagenase IV. Cells were extracellularly stained with anti‐CD3, anti‐CD4, anti‐CD25, anti‐F4/80, anti‐CD11b, anti‐CD80 and anti‐CD206 antibodies. Subsequently, the cells were fixed and permeabilized with Perm/Fix solution (eBioscience, San Diego, CA, USA) for 30 min. at 4°C. Finally, the cells were intracellularly stained with anti‐Foxp3 antibody (Biolegend, San Diego, CA, USA) at 4°C for 30 min. in the dark. Directly conjugated, isotype‐matched, rat antimouse Abs (Biolegend) served as controls. After washed with PBS twice, the pellet was resuspended in 400 μl PBS and analysed by the Calibur flow cytometer (BD).

### Immunofluorescence staining

Paraffin‐embedded tissues were cut into 5‐μm‐thick sections, the primary antibodies (Anti‐CD4, Abcam, Shanghai, China; Anti‐F4/80, Biolegend; Anti‐CD8, Abcam) were diluted to 1:50 in PBS and then treated sections at 4°C overnight. PE‐conjugated secondary antibody (red colour in the staining) was used to detect the primary antibody. Nuclei were counterstained with DAPI (Invitrogen, Carlsbad, CA, USA; blue colour). Antibody staining was detected using a fluorescent microscope (Olympus, Tokyo, Japan).

### Real‐Time quantified PCR

Total RNA was extracted with Trizol (Invitrogen). The expression levels of inflammatory cytokines, including IFN‐γ, TNF‐α, IL‐4, IL‐6, IL‐10 and IL‐17, were detected by Real‐time quantified PCR using SYBR green with a Bio‐Rad machine. The mRNA expression was normalized to β‐actin expression. Primers sequences are listed in Table 1.

**Table 1:** The primer sequences used for Real‐Time quantified PCR

Target gene	Primer sequence
IFN‐γ	F: 5′‐AGACAATCAGGCCATCAGCA‐3′
	R: 5′‐TGGACCTGTGGGTTGTTGAC‐3′
TNF‐α	F: 5′‐CCACCACGCTCTTCTGTCTA‐3′
	R: 5′‐GGTTTGCTACGACGTGGGC‐3′
IL‐4	F: 5′‐CAAACGTCCTCACAGCAACG‐3′
	R: 5′‐AGGCATCGAAAAGCCCGAAA‐3′
IL‐6	F: 5′‐ACAAAGCCAGAGTCCTTCAGAG‐3′
	R: 5′‐GCCACTCCTTCTGTGACTCC‐3′
IL‐10	F: 5′‐CAGTACAGCCGGGAAGACAAT‐3′
	R: 5′‐TTGGCAACCCAAGTAACCCT‐3′
IL‐17	F: 5′‐TCAATGCGGAGGGAAAGCTG‐3′
	R: 5′‐CCCACCAGCATCTTCTCGAC‐3′
β‐actin	F: 5′‐TGGACTTCGAGCAAGAGATG‐3′
	R: 5′‐GAAGGAAGGCTGGAAGAGTG‐3′

### Enzyme‐Linked Immunosorbent Assay (ELISA)

The concentration of IFN‐γ, TNF‐α, IL‐4, IL‐6, IL‐10 and IL‐17 in the serum and colorectal tissues of mice was detected by ELISA Kit (eBioscience) according to the manufacturer's instructions. Absorbance was read at 450 nm within 20 min. using an ELISA reader (Bio‐Rad Laboratories, Hercules, CA, USA).

### Ear weight and thickness measurements and analysis

Ear thickness was measured using a digital caliper, followed by an 8‐mm punch biopsy to ascertain changes in ear weight. The biopsies were weighed individually using an analytical balance (Sartorius, Göttingen, Germany).

### Psoriasis severity evaluation

To evaluate the severity of inflammation in the ears and neck skin of mice, we developed an objective scoring system 32 based on the clinical Psoriasis Area and Severity Index (PASI). Erythema, scaling and thickening were scored independently on a scale from 0 to 4: 0, none; 1, slight; 2, moderate; 3, marked; or 4, very marked.

### Statistical analysis

Data are presented as averages ± S.D. and were analysed by student's *t*‐test using GraphPad prism 6. *P *≤ 0.05 was considered significant.

## Results

### IL‐35 recombinant protein effectively relieves pathological features in DSS‐induced acute colitis mouse model

**Figure 1:**
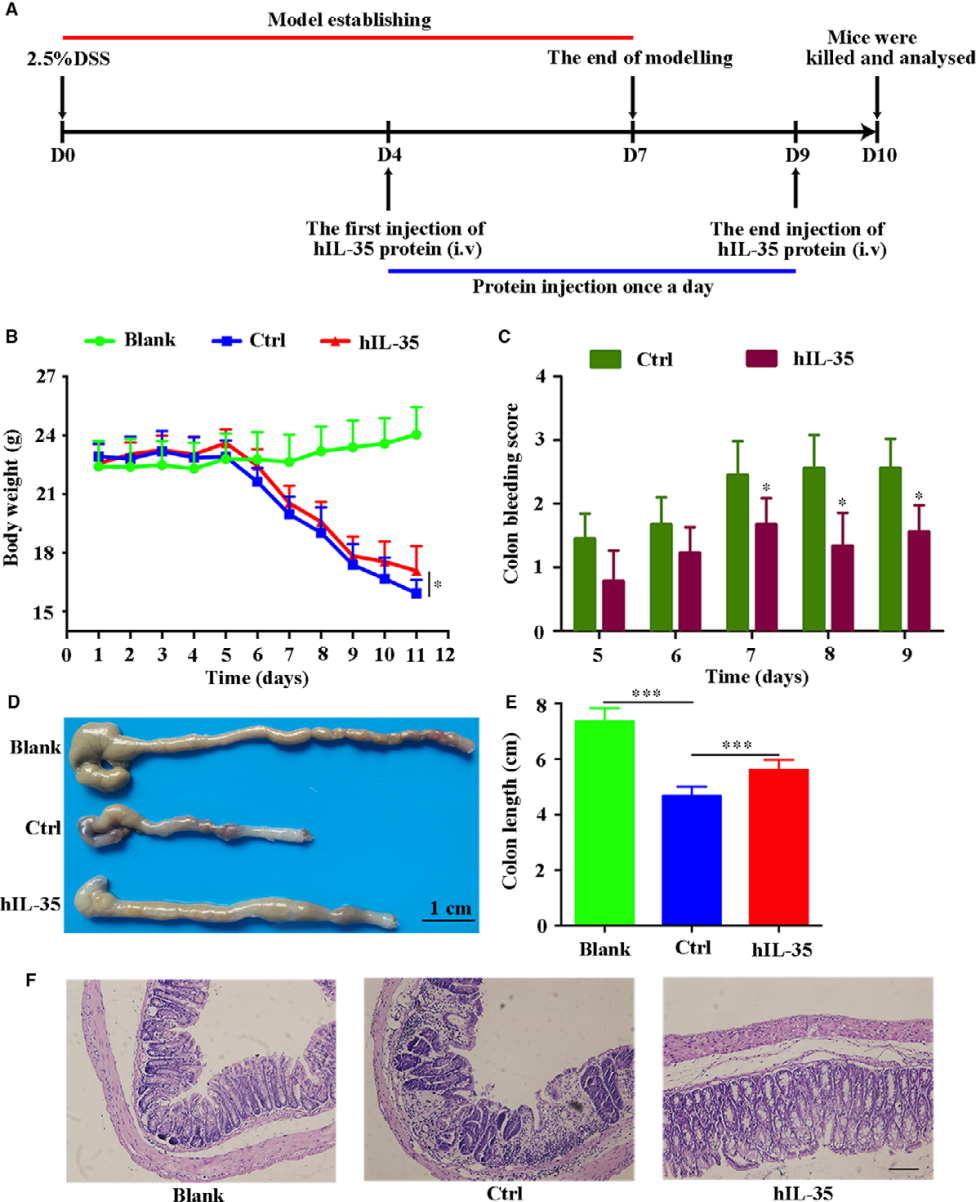
IL‐35 recombinant protein effectively relieves pathological features in DSS‐induced acute colitis mouse model. (**A**) The DSS‐induced process and dosage regimen of IL‐35 recombinant protein. (**B**) Mice body weight of different groups was measured everyday (*n* = 8 per group). (**C**) Colon bleeding score was assessed as the combined score of faecal consistency and blood in the stool throughout the DSS treatment and recovery period (scored as 0–4). (**D** and **E**) Representative photographs of mouse colon in different groups (**D**) and the length of the colon were measured (**E**). (**F**) H&E staining of mice colon sections. Scale bar, 100 μm. Data are shown as means ± S.D. Statistical significance was assessed by unpaired, two‐tailed Student's *t*‐test (***P <* *0.001, *P <* *0.05).

To determine whether IL‐35 recombinant protein exerts potential therapeutic effects in acute colitis mouse model, we treated DSS‐induced acute colitis mice with human IL‐35 recombinant protein. The DSS‐induced process and dosage regimen of IL‐35 recombinant protein are illustrated in Fig. 1A. The mice were killed, and relevant indicators were analysed on day 10 after the first injection of DSS. The data indicated that IL‐35 could alleviate body weight loss to a certain extent (Ctrl: 16.33 ± 1.36 *versus* hIL‐35: 17.46 ± 1.64) compared with control group (Fig. 1B). In addition, mice in IL‐35 group had less blood in the stool and the survival status was better (Fig. 1C). What is more, the colon length in IL‐35 group was longer than (Ctrl: 4.67 ± 0.33 *versus* hIL‐35: 5.61 ± 0.36) that in control group (Fig. 1D and E). H&E staining also showed that the basic structure of colorectal tissues was seriously damaged in control group, accompanying with infiltration of inflammatory cells into the lamina propria (Fig. 1F). In contrast, the colon of IL‐35 group was relatively integrated and the infiltration of lymphocytes was decreased significantly. These results indicated that IL‐35 can effectively relieve pathological features in DSS‐induced acute colitis mouse model.

### IL‐35 recombinant protein regulates the infiltration of macrophages in colonic lesions

**Figure 2:**
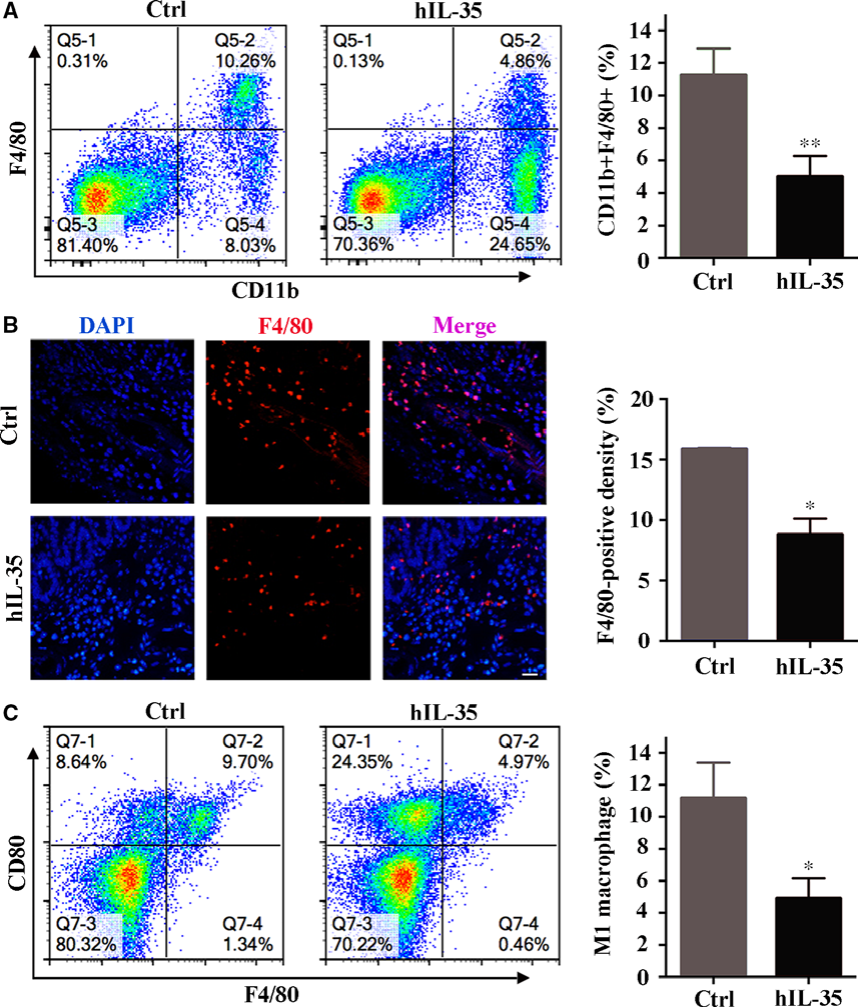
IL‐35 recombinant protein regulates the infiltration of macrophages in colonic lesions. (**A**) Left: Flow cytometry analysed the proportion of macrophages (CD11b^+^F4/80^+^) in control and IL‐35 group (*n* = 8 per group). Right: quantitative analysis the proportion of macrophages in different groups. (**B**) Left: immunofluorescence analysis of macrophages in colon sections. F4/80 was detected in red and DAPI (for nuclei staining) was blue. Right: quantitative analysis of F4/80^+^ cells. (**C**) Flow cytometry analysed the proportion of M1 macrophages in control and IL‐35 group (Left) and corresponding statistical data were showed (Right). Scale bar, 50 μm. Data are shown as means ± S.D. Statistical significance was assessed by unpaired, two‐tailed Student's *t*‐test (**P <* *0.01, *P <* *0.05).

Inflammatory bowel disease is an autoimmune disease caused by the abnormal interactions between a series of immune cells, cytokines and antigens. The mucosal damages related to activated macrophages and T cells were considered as the main cause of IBD pathological changes 33, 34. Consequently, we investigated the proportion of macrophages in colorectal tissues by flow cytometry and immunofluorescence staining. The results showed that IL‐35 recombinant protein was able to down‐regulate the macrophage infiltration (Ctrl: 15.83 ± 0.11 *versus* hIL‐35: 8.78 ± 1.33) compared with control group (Fig. 2A and B).

Pro‐inflammatory macrophages (M1) have been identified as key contributor in the pathological process of IBD. Our results showed that about 90% F4/80‐positive cells expressed M1 macrophages marker CD80, and M1 macrophages (F4/80^+^/CD80^+^) were significantly inhibited (Ctrl: 11.16 ± 2.22% *versus* hIL‐35: 4.89 ± 1.26%) in the IL‐35 group (Fig. 2C). Together, IL‐35 recombinant protein may reduce the development of IBD through decreasing the number of pro‐inflammatory macrophages in colonic lesions.

### IL‐35 recombinant protein regulates the infiltration of T cells in colonic lesions

**Figure 3:**
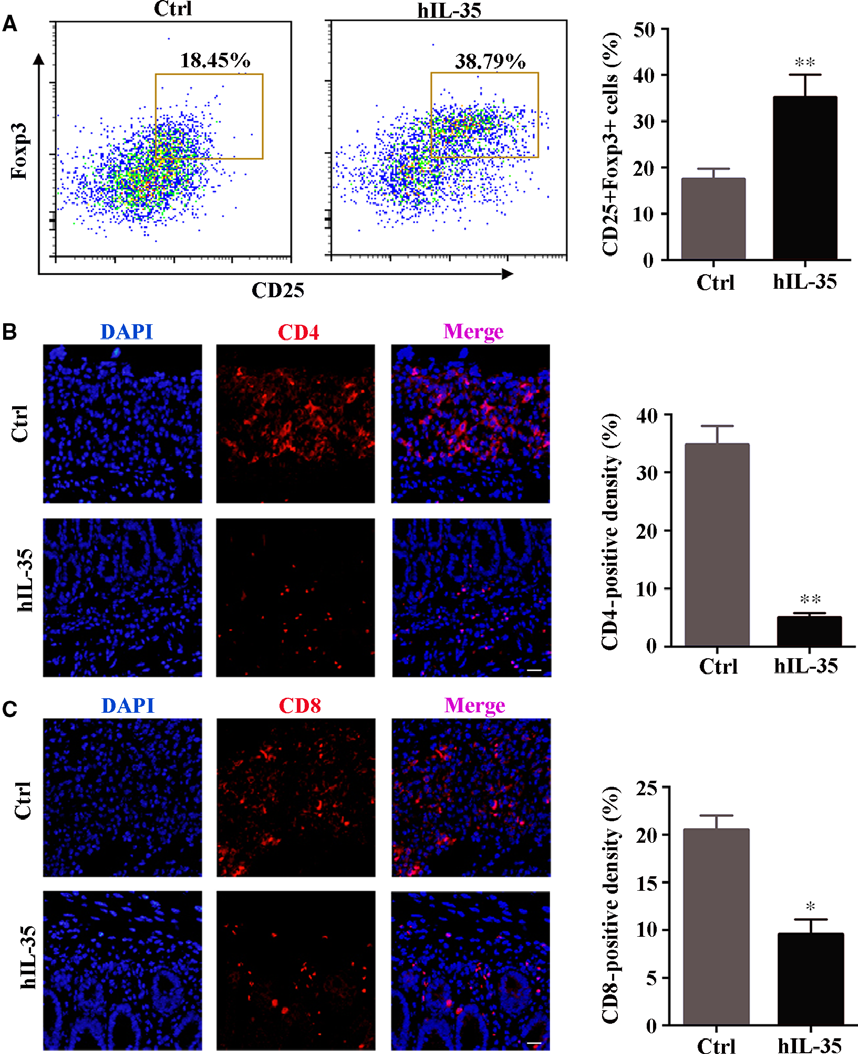
IL‐35 recombinant protein regulates the infiltration of T cells in colonic lesions. (**A**) Left: flow cytometry analysed the proportion of Treg cells (CD25^+^Foxp3^+^) in control and IL‐35 group (*n* = 8 per group). Right: quantitative analysis the proportion of Treg cells in different groups. (**B**) Left: immunofluorescence analysis of CD4^+^ T cells in colon sections. CD4 was detected in red and DAPI (for nuclei staining) was blue. Right: quantitative analysis of CD4^+^ T cells. (**C**) Left: immunofluorescence analysis of CD8^+^ T cells in colon sections. CD8 was detected in red and DAPI (for nuclei staining) was blue. Right: quantitative analysis of CD8^+^ T cells. Scale bar, 50 μm. Data are shown as means ± S.D. Statistical significance was assessed by unpaired, two‐tailed Student's *t*‐test (**P <* *0.01, *P <* *0.05).

The unbalanced cellular immune responses seem to be mainly responsible for the initiation and development of the inflammation in IBD. These adaptive cellular responses involve activation of T helper (Th) lymphocytes and suppression of the activity of T regulatory (Treg) cells 35. We examined the subtypes of T lymphocytes to investigate whether IL‐35 recombinant protein also acted on T cells to play its regulative role. Our data indicated that the ratio of Treg cells in colorectal tissues was significantly up‐regulated after the treatment of IL‐35 (Ctrl: 17.43 ± 2.31% *versus* hIL‐35: 35.09 ± 4.97%), indicating that IL‐35 recombinant protein could effectively induce regulatory T cells to inflammatory site and inhibit the inflammation (Fig. 3A). Then we detected CD4^+^T cells and CD8^+^T cells in colorectal tissues by immunofluorescence staining (Fig. 3B and C) and found that IL‐35 recombinant protein could significantly decrease the infiltration of CD4^+^T cells (Ctrl: 34.70 ± 3.33% *versus* hIL‐35: 4.97 ± 0.84%) and CD8^+^T cells (Ctrl: 20.47 ± 1.55% *versus* hIL‐35: 9.54 ± 1.58%). These data indicated that IL‐35 recombinant protein may relieve the development of IBD through the regulation of immune cells.

### IL‐35 recombinant protein regulates inflammatory cytokines in acute colitis mouse model

**Figure 4:**
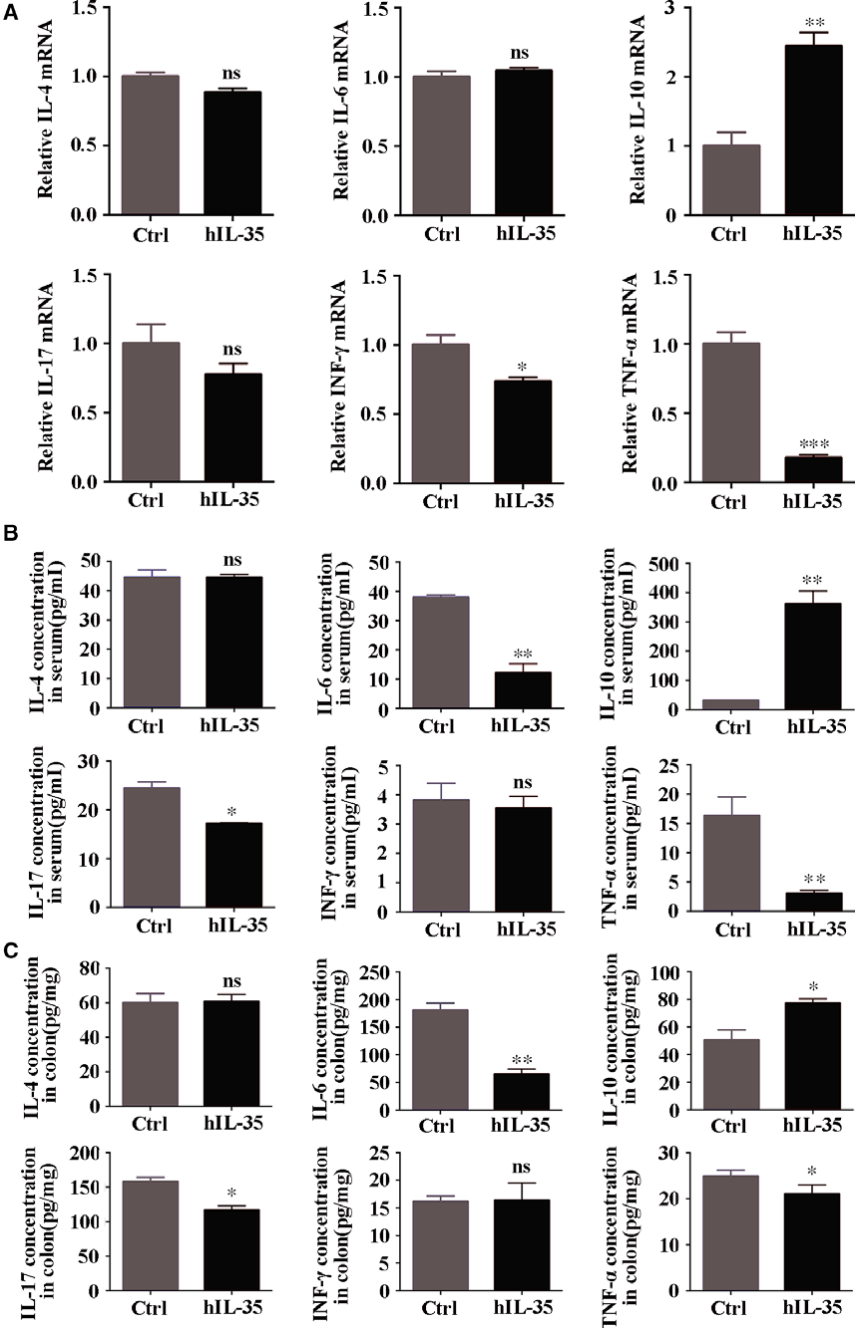
IL‐35 recombinant protein regulates inflammatory cytokines in acute colitis mouse model. (**A**) Quantitative RT‐PCR analysis of IL‐4, IL‐6, IL‐10, IL‐17, IFN‐γ and TNF‐α messenger RNA (mRNA) expression in colorectal tissues (normalized to β‐actin). (**B**) Determination of the IL‐4, IL‐6, IL‐10, IL‐17, IFN‐γ and TNF‐α concentration in serum by ELISA. (**C**) Determination of the IL‐4, IL‐6, IL‐10, IL‐17, IFN‐γ and TNF‐α concentration in colorectal tissues lysate by ELISA. Data are shown as means ± S.D. Statistical significance was assessed by unpaired, two‐tailed Student's *t*‐test (***P <* *0.001, **P <* *0.01, *P <* *0.05).

The dysregulation of anti‐inflammatory and pro‐inflammatory factors plays an important role in the further deterioration of the inflammatory bowel disease 36. We first detected the changes of these cytokines at the mRNA level. After the treatment of IL‐35 recombinant protein, the expression of IL‐10 was significantly up‐regulated (Ctrl: 1.00 ± 0.20 *versus* hIL‐35: 2.44 ± 0.19), while the pro‐inflammatory cytokines such as TNF‐α (Ctrl: 1.00 ± 0.09 *versus* hIL‐35: 0.18 ± 0.02) and IFN‐γ (Ctrl: 1.00 ± 0.07 *versus* hIL‐35: 0.74 ± 0.03) were decreased in colorectal tissues (Fig. 4A). We further detected the concentration of these cytokines by ELISA. Our data demonstrated that the concentration of IL‐10 in mice serum treated with IL‐35 recombinant protein was increased significantly compared with the control group (Ctrl: 32.03 ± 0.02 *versus* hIL‐35: 361.73 ± 44.09). In contrast, pro‐inflammatory cytokines IL‐6 (Ctrl: 37.84 ± 0.84 *versus* hIL‐35: 12.21 ± 3.06), IL‐17 (Ctrl: 24.43 ± 1.33 *versus* hIL‐35: 17.19 ± 0.14) and TNF‐α (Ctrl: 16.35 ± 3.17 *versus* hIL‐35: 3.07 ± 0.48) were significantly decreased (Fig. 4B). As the colon is the main pathogenic site of DSS‐induced acute colitis model, we also examined the protein changes of cytokines in colorectal tissues and the variation trend of cytokines was consistent with those in serum (Fig. 4C). Based on the above results, we speculated the anti‐inflammatory effects of IL‐35 recombinant protein may result from the comprehensive effects which promote the secretion of anti‐inflammatory factor IL‐10 and simultaneously suppress the secretion of pro‐inflammatory cytokines such as IL‐6, IL‐17 and TNF‐α.

### IL‐35 recombinant protein can improve pathological phenotype in K14‐VEGF‐A‐tg psoriasis **model**

**Figure 5:**
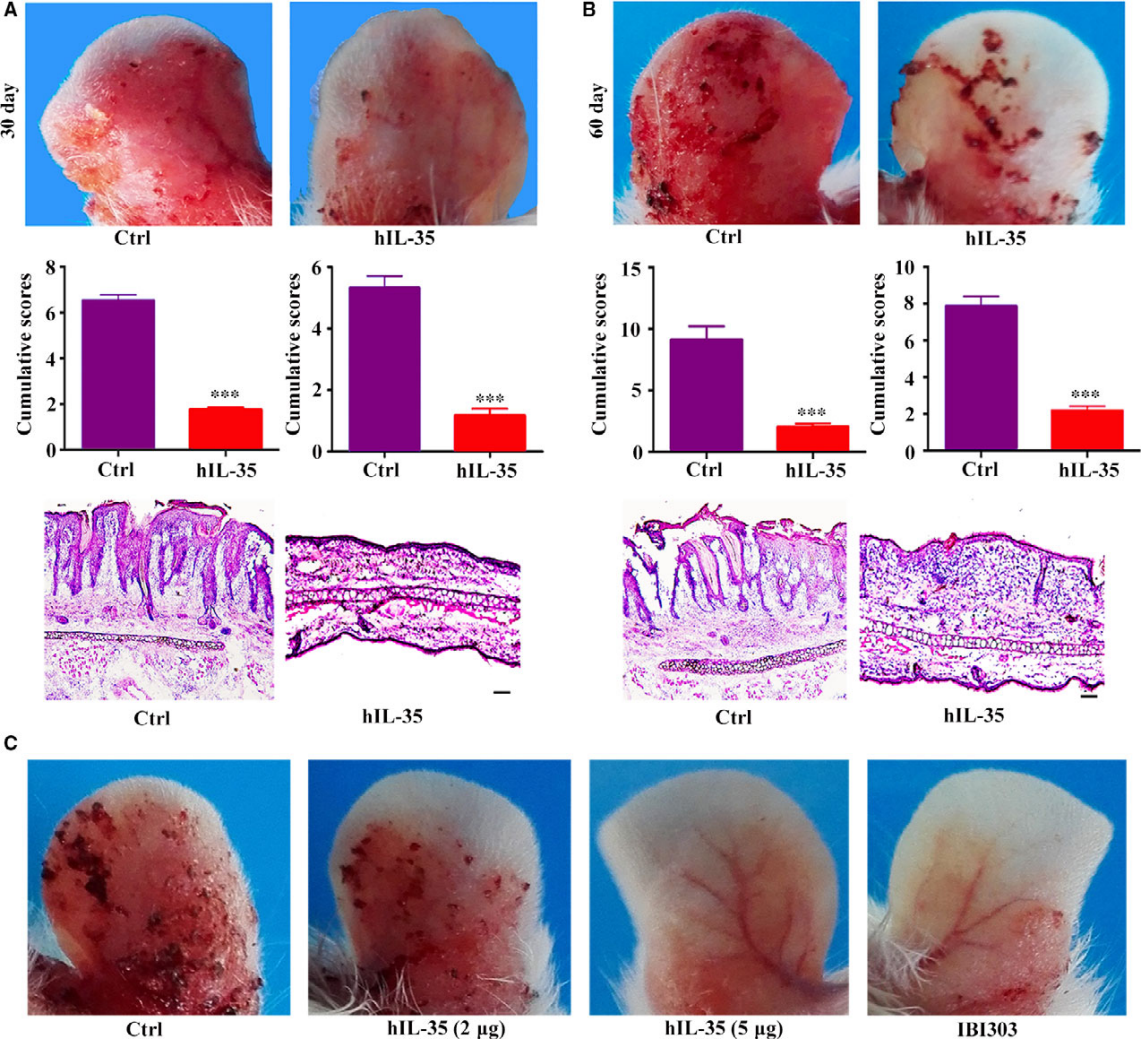
IL‐35 recombinant protein can improve pathological phenotype in K14‐VEGF‐A‐tg psoriasis model. (**A**) Upper: mice (*n* = 8 per group) psoriatic symptoms were assessed *via* macroscopic observation at day 30 (indicated as 30 days) after the treatment period. Middle: cumulative scores (erythema + scaling + thickness) were calculated and depicted. Lower: H&E staining of ear skin at 30 days. (**B**) Upper: mice psoriatic symptoms were assessed *via* macroscopic observation at day 60 (indicated as 60 days) after the treatment period. Middle: cumulative scores (erythema + scaling + thickness) were calculated and depicted. Lower: H&E staining of ear skin at 60 days. (**C**) Representative photographs of mice psoriatic symptoms in different groups treated with PBS, IL‐35 recombinant protein or IBI303. Scale bar, 100 μm. Data are shown as means ± S.D. Statistical significance was assessed by unpaired, two‐tailed Student's *t*‐test (***P <* *0.001, *P <* *0.05).

Based on our previous results that IL‐35 gene therapy could significantly alleviate the pathogenesis of K14‐VEGF‐A‐tg psoriasis mice 27, we further investigated whether IL‐35 recombinant protein shows therapeutic potential on K14‐VEGF‐A‐tg psoriasis mice. Mice were killed on the 30th and 60th day after the last IL‐35 recombinant protein treatment, and samples were collected for subsequent analysed. On the 30th day, mice in the control group had obvious redness, scales and other psoriasis‐like lesions accompanied by severe ear thickening and swelling. After the treatment of IL‐35 recombinant protein, the ear injury was significantly alleviated. We evaluated the two groups by PASI scoring system and H&E staining which also came to the same conclusion that IL‐35 recombinant protein could significantly relieve the pathological status of the transgenic mice (Fig. 5A). Although deterioration of lesions on the ear tissues was observed in the IL‐35 group on 60 days after the treatment, it was still significantly better than the control group (Fig. 5B).

TNF‐α inhibitors are highly effective for the treatment of psoriasis 37. We have confirmed that TNF‐α monoclonal antibody IBI303 could achieve good effects in the treatment of psoriasis by anti‐inflammation and inhibiting angiogenesis 31. However, the degree of immunosuppression induced by blocking TNF‐α can lead to adverse events 38, 39. Thus, we compared the therapeutic effects of IL‐35 recombinant protein with IBI303. We found that IL‐35 recombinant protein was able to reach comparable results as the TNF‐α monoclonal antibody did in regard to the treatment of psoriasis (Fig. 5C).

### IL‐35 recombinant protein can slow down the pathological process in the IMQ‐induced psoriasis mouse model

**Figure 6:**
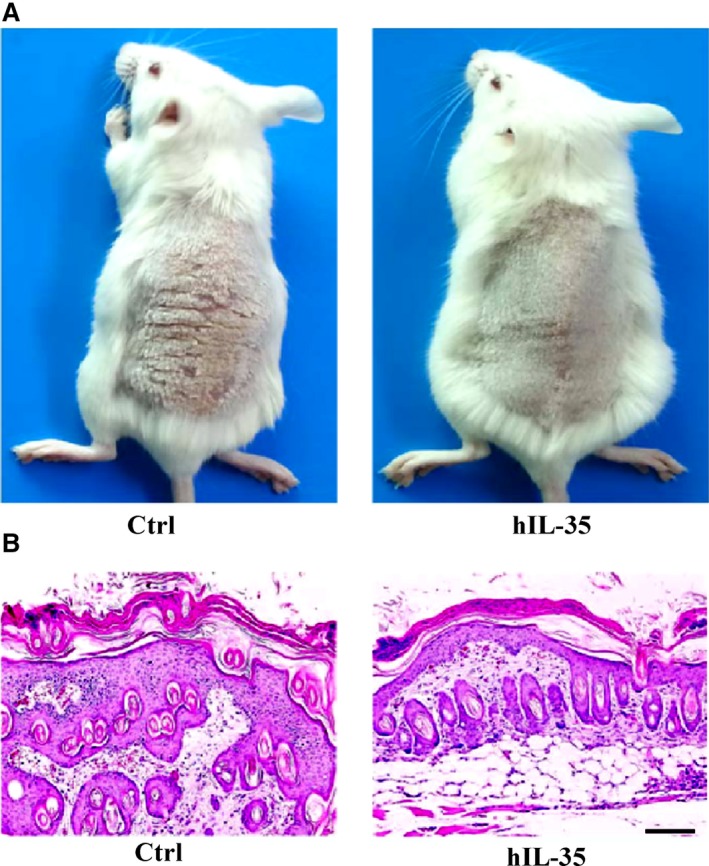
IL‐35 recombinant protein can slow down the pathological process in the IMQ‐induced psoriasis mouse model. (**A**) Representative photographs of the skin tissue in IMQ‐induced psoriasis mouse with or without IL‐35 recombinant protein treatment. (**B**) H&E‐stained skin sections from IMQ‐treated mice showed increased epidermal thickening in control group compared with IL‐35 group. Scale bar, 100 μm.

To further demonstrate the therapeutic effects of IL‐35 recombinant protein in psoriasis, we used IMQ to establish a chemical‐induced psoriasis mouse model for prophylactic treatment with IL‐35 recombinant protein. We killed the mice on the day after the last treatment and photographed the dorsal skin. We found that the control group had greater inflammation and flaking of the skin which continued to increase in severity until the end of the experiment (Fig. 6A). Analysis of H&E‐stained skin sections from IMQ‐induced mice showed increased epidermal thickening in the control group than that in IL‐35 group (Fig. 6B). These results suggested that IL‐35 recombinant protein could effectively weaken the inflammatory process in the IMQ‐induced psoriasis skin mouse model as well.

## Discussion

IL‐35 is a new member of the IL‐12 family involved in immune regulation of multiple diseases and tumours 40. Studies have shown that IL‐35 could inhibit the development of inflammatory bowel disease and psoriasis by means of the gene therapy. Both IBD and psoriasis are immunity‐related diseases involved in the dysregulation of inflammatory cytokines and immune cells 41, 42. They share some common inflammatory and regulatory pathways such as TNF‐α and IL‐17 signalling as well as NFκB activation. Drugs that target TNF‐α (Infliximab), IL‐12/23 (Ustekinumab) and IL‐17 have showed potential therapeutic effect in both IBD and psoriasis 37, 43. However, there is still an urgent need for more targeted therapies. In this study, we established the acute colitis and psoriasis mouse models to investigate the anti‐inflammatory effects of IL‐35 recombinant protein and carried out the preliminary study to investigate its immune regulation mechanism. Our results indicated that IL‐35 recombinant protein could reverse inflammatory bowel disease and psoriasis through regulation of inflammatory cytokine secretion and immune cells infiltration.

IBD, a recurring and uncontrollable colorectal inflammation, is one of the important inducements of colorectal cancer 40. Studies have shown that the therapeutic efficacy on EBI3‐deficient mice would be significantly inhibited in the IBD mouse model 25, indicating the critical role of IL‐35 in immunoregulation. Our results indicated that IL‐35 recombinant protein could significantly inhibit the inflammatory phenotypes of model mice.

A number of clinical results showed that compared with normal people, the colorectal tissues of ulcerative colitis patients manifested the high macrophage infiltration. With the disease progressing, the ratio of macrophage infiltration also raised gradually 19, 44, 45. Consistent with our previous results that IL‐35 gene therapy could significantly alleviate the pathogenesis of psoriasis mice, our present data showed that IL‐35 recombinant protein could significantly decrease the number of macrophages in the colorectal tissues. In addition, the immunofluorescence staining of colorectal tissues showed that IL‐35 recombinant protein could significantly inhibit the infiltration of CD4^+^T cells and CD8^+^T cells. Treg cells are constitutively present in the intestinal mucosa and function in the suppression of immune responses through inhibiting the proliferation of effector T cells. Previous studies indicated that Treg cells from mice deficient in either EBI3 or p35 have decreased regulatory activity, suggesting IL‐35 is required for optimal Treg cell function 5. Thus, we detected the changes of Treg cells in colorectal tissues by flow cytometry. Consistent with previous results that IL‐35 could induce the generation of Treg cells, the proportion of Treg cells was significantly up‐regulated after the treatment of IL‐35 recombinant protein.

Intestinal inflammation was mainly mediated by the immune response of Th1, Th2 and Th17 cells 46, 47, 48. The interactions between these and other cells present in the inflamed mucosa are mediated by various cytokines including both pro‐inflammatory and anti‐inflammatory cytokines. It is widely known that IL‐10 can antagonize Th1 cytokines and stabilize intestinal mucosal immune balance, serving as an important anti‐inflammatory cytokines which plays a protective role in the development of IBD 36, 49. Our data demonstrated that IL‐10 was up‐regulated after the treatment of IL‐35 recombinant protein. In contrast, several pro‐inflammatory cytokines including IL‐6, IL‐17 and TNF‐α were decreased in serum and colorectal tissues. Although a complex network of interactions exists between inflammatory cytokines and immune cells, it is apparent that IL‐35 plays an essential role in the balance between Th17 cells and Treg cells. On one hand, IL‐35 is able to induce the generation of Treg cells that produce IL‐35 (iTr35 cells), and more iTr35 cells further secrete more IL‐35. On the other, IL‐35 potently inhibits Th1 and Th17 cells through the expansion of Treg cells as well as IL‐10 secretion. Moreover, IL‐10 plays a role in the suppression of macrophage functions, further resulting in the decreasing IL‐6. Together, IL‐35 may induce the generation of the anti‐inflammatory milieu *via* regulation of inflammation‐associated cytokines secretion.

Psoriasis is a chronic, immune‐mediated disorder that affects millions of people and the pathophysiology is complex and dynamic, involving skin cells and immune cells. A large amount of evidence has defined the essential role of the immune system and its interactive network of leucocytes and cytokines in psoriatic pathogenesis 50. Psoriatic lesions are highly infiltrated with immune cells, and pro‐inflammatory cytokines produced by these cells such as TNF‐α, IL‐17 and IL23 have been linked to the pathogenesis of psoriasis. The anti‐inflammatory potential of IL‐35 gene therapy in psoriasis has been investigated in our previous study. In our present study, we continued to investigate whether IL‐35 recombinant protein could achieve the same therapeutic effect in K14‐VEGF transgenic psoriasis mouse model and IMQ‐induced psoriasis mouse model. We found that IL‐35 recombinant protein could attenuate severe psoriasis‐like symptoms in both psoriasis mouse models. In our previous study, we have demonstrated that four‐cycle treatment of anti‐TNF‐α monoclonal antibody (IBI303) could reverse psoriasis through dual inhibition of inflammation and angiogenesis. Intriguingly, lower dose of IL‐35 could achieve a therapeutic effect similar to what TNF‐α monoclonal antibody did in psoriasis (about a total of 50 μg hIL‐35 compared to 3.75 mg IBI303 per mouse). The data indicated that IL‐35 recombinant protein possessed potential anti‐inflammatory effects and was expected to become a promising candidate drug for the treatment of inflammatory diseases in the future.

Taken together, our study found that IL‐35 recombinant protein could slow down the pathogenesis of acute colitis mouse model mainly by reducing the macrophages, CD4^+^T and CD8^+^T cell infiltration as well as promoting the proportion of Treg cells. What is more, IL‐35 could also achieve the effects of inhibiting of inflammation through the regulation of multiple inflammation‐associated factors. In addition, in the psoriasis mouse model, lower dose of IL‐35 recombinant protein can achieve long‐term therapeutic effects as TNF‐α monoclonal antibody did. IL‐35 recombinant protein showed good therapeutic effects in acute colitis and psoriasis mouse model, indicating that IL‐35 recombinant protein had a strong anti‐inflammatory ability. Our results highlighted the important role of IL‐35 in the development and pathogenesis of IBD and psoriasis, and these discoveries might translate into future therapies for immune diseases.

## Author contributions

Y.W., Y.M. and J.Z. involved in conception and design, collection and assembly of data, data analysis and interpretation and manuscript writing; G.S., L.C., Y.L., Y.L. and X.Z. involved in provision of study material, data analysis and interpretation; Y.Z., X.C., J.D., X.S., L.D., Y.Y., S.Z.D.Y. and Y.W. involved in provision of study material, conception and design and assembly of data; H.D. involved in conception and design, manuscript writing, financial support and final approval of manuscript.

## Conflict of interest

The authors indicate no potential conflict of interest.
